# Use of Complementary and Alternative Medicine among People with Type 2 Diabetes in Taiwan: A Cross-Sectional Survey

**DOI:** 10.1155/2011/983792

**Published:** 2010-09-08

**Authors:** Hsiao-yun Annie Chang, Marianne Wallis, Evelin Tiralongo

**Affiliations:** ^1^Fooyin University, 151, Chinhsueh Rd., Ta-liao, Kaohsiung 83101, Taiwan; ^2^Research Centre for Clinical and Community Practice Innovation, Griffith University, Southport QLD 4222, Australia; ^3^Research Centre for Clinical and Community Practice Innovation and Gold Coast Health Service District, Griffith University, Southport QLD 4215, Australia; ^4^School of Pharmacy, Griffith University, Gold Coast QLD 4222, Australia

## Abstract

Research into CAM use by people with diabetes is limited. This study explored CAM use among patients who attend diabetic clinics for followup treatment. Special attention was paid to patients' changing patterns of CAM use before and after diagnosis with Type 2 diabetes, their experience of CAM use, and their management of CAM use with conventional medicines. A retrospective cross-sectional survey (*n* = 326) was undertaken in three census regions in Taiwan, including metropolitan, urban, and rural areas in 2006-7 (87.4% response rate). Participants reported extensive use of CAM with conventional medicines. The prevalence of CAM use was 22.7% before and 61.0% after diagnosis with Type 2 diabetes with nutritional supplements being the most commonly used CAM before and after diagnosis. However, the disclosure rate of CAM use to healthcare professionals remained low (24.6%), and lack of knowledge about CAM ingredients was common (63.4%). Awareness of the widespread use of CAM by people with Type 2 diabetes is crucial for healthcare professionals. The self-administration of both conventional medicines and CAM without disclosure of CAM use to healthcare professionals may result in ineffective diabetes management and adverse effects. CAM information needs to be incorporated into clinical practice and patient and professional education.

## 1. Introduction

Global estimates of the prevalence of diabetes for 2010 is around 6.4%, affecting 285 million adults, and will increase to 7.7%, and 439 million adults by 2030 [1]. Much of this increase in diabetes will occur in Asia, such as India and China. With Taiwan being part of the Asia Pacific region, the prevalence of diabetes is high at around 4.5% [2]; this disease is, in fact, emerging as a major health issue in Taiwan. The presence of such a chronic, debilitating, and possibly painful illness has been identified as a reason why patients seek out CAM [3]. In addition, Chang et al. [4] highlighted that the prevalence of CAM use among diabetic populations worldwide varies widely, depending on the definition of CAM and the survey design used by researchers. The prevalence ranged from 17% in a UK study [5] to 72.8% in the USA [6] with an average of 45.5% of participants in the studies reporting the use of some form of CAM. Although evidence is mounting in support of the use of various CAM to treat a wide variety of complications of diabetes mellitus [7], whether patients with Type 2 diabetes actually use CAMs with known benefits in the management of diabetes is largely unknown. Especially, the patterns of CAM use among the Type 2 diabetes population are largely unknown, and no relevant study has been conducted among patients with Type 2 diabetes in Taiwan. 

Research into the reasons for CAM use by people with diabetes is also limited. Some researchers have identified the growth of CAM use in other patient populations as being associated with the perceived limitations of the medical paradigm and the apparent failure of conventional medicine to treat and/or cure chronic illness and catastrophic diseases [8]. However, Coulter and Willis [9] suggest that the growth in CAM use may be related to general societal changes. As social change and globalization accelerate, faith in the ability of medical science to solve the problems of human diseases has declined. This change within society might be interpreted as part of the ascendancy of patient self-empowerment [10]. This view suggests that patients seek out CAM because they believe it offers them more personal autonomy and control over their healthcare decisions [11, 12]. However, several researchers have found there are more complex reasons associated with philosophical congruence related to CAM use such as patients' values, worldviews, spiritual/religious philosophies, beliefs, or culture in relation to the nature and meaning of health and illness [13]. Thus, understanding the reasons for patients' CAM use is important as it will help healthcare professionals to understand the factors that underpin patients' beliefs and attitudes towards their health care. This is the case for any patient population including people with Type 2 diabetes.

The majority of patients use CAM in conjunction with conventional medicine, not as an alternative. Some studies investigated the issue of communication of CAM use with conventional healthcare professionals, only some referred to diabetic patients. Egede and his colleagues [14] found fewer than 40% of Americans with diabetes who used CAM disclosed this information to their physicians. Little is known about the disclosure rates among Taiwanese diabetes patients and a recent study showed that 35.4% had discussed CAM use with their psychiatrists [15]. However, the literature offers little discussion of the reasons for this limited disclosure of CAM use and the apparent communication gap between patients and healthcare professionals. This lack of discussion may indicate a deficiency in the relationship between patient and healthcare professional which could have negatively impact on patient care and health outcomes. In addition, most previous studies of CAM use among people with diabetes have used data derived from either a medical expenditure survey or from health insurance claims in the USA. Thus, these studies mainly focused on CAM users' characteristics, but have not explained patients' attitudes, motivations, and knowledge about CAM use. 

Research into the extent of CAM use, why and how it is used, and disclosure of use to healthcare professionals is vital as results could help to improve communication between healthcare professionals and patients and assist in planning better self-management strategies for patients. The purpose of this study was to survey people with Type 2 diabetes at diabetic clinics in order to identify patterns of CAM use before and after diagnosis, their experience of CAM use, and their concomitant use of CAM with conventional treatments. 

## 2. Methods

### 2.1. Study Design and Sample

A retrospective cross-sectional survey, conducted as a structured interview, was undertaken between July 2006 and February 2007, in conventional (Western) hospitals in three regions in Taiwan: Taipei (major metropolitan area), Kaohsiung (regional area), and Pin-tong (rural area). In order to conduct a representative survey, Moser and Kalton's formula [16] was used to determine the sample size and the acceptable amount of sampling error was set at three percent (0.03) for this study. According to this formula, given that the sampling population (people with Type 2 diabetes) from these three regions was around 4000, a sample size of at least 271 participants were necessary to give significant results. Three clinics recruited respondents at the same time and it was difficult to control the exact sample size during parallel recruiting. Finally, a total of 373 respondents who were 18 years or older and spoke one of the following languages: Mandarin, Fujian, or Hakka were invited to participate in this study, with 47 patients declining consent. A total of 326 participants completed face-to-face structured interviews (87.4% response rate). 

A two-stage sampling design involving clustering and simple sampling was used in the selection process. Three hospitals were selected as a cluster. All respondents within each cluster were grouped as they entered clinics at the same time (morning, afternoon, and evening). The appointment number was the sampling frame and then a simple random sampling technique was used to draw a sample of the desired size. 

The characteristics of the sample were similar to the diabetes population distribution published by the Taiwanese Bureau of Health Census in 2005 indicating that the sample population in this study reasonably represented the Taiwanese diabetes population. The human research ethics committees of one of the hospitals in Taiwan and an Australia university approved the study. The other hospitals gave permission based on these ethics reviews. Written informed consent to participate in the study was obtained before proceeding with the interview.

### 2.2. Survey Instrument

The survey instrument was divided into three sections: demographic characteristics, pattern of CAM use, and experience of CAM use. Demographic data were collected in relation to age, gender, education, income, duration of disease, and frequency of clinic visits. In the second section, data were collected about the use of 14 specific CAM modalities. These modalities were chosen following a literature review, and they represent the modalities most frequently reported in previous studies undertaken in Taiwan and internationally. The definition of the term CAM used in this study encompasses all the domains proposed by the National Centre for Complementary and Alternative Medicine [2], including whole medicine systems, mind-body medicine, biologically based practices, manipulative and body-based practices, and energy medicine. For example, for biologically based practices, nutritional supplements, diet modifications, Chinese herbal medicines, and non-Chinese herbal medicines were chosen. A cardboard prompt list with the names and descriptions of the various CAM modalities was used to stimulate participants' memory during the interviews (see Table 1). Then, the interviewer read the following statement verbatim to each participant: “Did you ever use the following therapies such as the treatments shown on this card before diagnosis with diabetes? Have you used any of them since diagnosis?” Participants who responded affirmatively were asked to indicate the specific treatment, and the reasons for its use. The third section of the survey, based on a review of Taiwanese and English language literature, focused on patterns of CAM use including: the reasons for CAM use, factors influencing the decision to use CAM, the administration of CAM with conventional treatments, the disclosure of CAM use to healthcare professionals, and the reasons for not using CAM or ceasing CAM.

The content validity of the survey instrument was established by a panel of experts. Three academic professionals and two nurse educators who were expert in CAM, nursing, research methods, or diabetes evaluated each survey item and also considered whether all the items adequately measured the dimensions of the content domain. Only a few items, which were identified as not adequately presented, were retained, revised, or replaced following discussion. However, most of items were determined to be appropriate for assessing each content domain. Because the survey instrument was originally written in English and was then translated into Chinese, the reliability of the translation of the survey instrument content was ensured by using back-translation. The original and back-translated versions were compared for equivalence in meaning by a group of bilingual experts. A pilot study was conducted before the onset of the main study. The survey conditions of this pilot study were similar to the actual survey and people who participated in the pilot study were excluded from the main study.

### 2.3. Data Collection Procedures

Research assistants (RAs) randomly selected potential participants from the list of appointments. Clinic staff assisted with recruitment of potential participants. After determining that potential participants met the inclusion criteria, the information sheet and the consent form were discussed and signed. The RA performed the structured interviews and recorded all answers on the survey instrument.

### 2.4. Statistical Analyses

The data were scanned for completeness, and responses were coded and entered into the computer program SPSS for Windows Version 14.0. The data were examined for outliers, and errors in coding and data entry were corrected. Demographic and clinical data characterizing the sample were summarized through descriptive statistical procedures. In order to identify whether people were more likely to use CAM, and/or use it more frequently/use more modalities, after diagnosis with Type 2 diabetes, two ways of analyzing before and after diagnosis usage were employed. First, the proportions using each CAM modality were compared using the McNemar test and second, the mean numbers of CAM modalities used in each group were compared using the Wilcoxon Signed Ranks Test. The level for statistical significance for all analyses was set at a minimum of *P* < .05.

## 3. Results

### 3.1. Characteristics of Sample

Characteristics of the sample including demographic and clinical information are presented in Table 2. The majority of participants were female, middle-aged, with at least a high school education, had a household annual income range of US$10,001–30,000 (NT$330,001–990,000) were diagnosed with Type 2 diabetes less than ten years before the study, and visited the diabetes clinic monthly. When the key characteristics of gender and age were compared between the sample and the diabetic population of Taiwan the results indicated that there were no statistically significant differences between the sample and the population (Gender: (*χ* ^2^(1) = 0.14, *P* = .84); Age (*χ* ^2^(4) = 2.68, *P* = .61).

### 3.2. Prevalence and Patterns of Change of CAM Use before and after Diagnosis of Type 2 Diabetes

Of the 326 participants, 22.7% (*n* = 74) reported using CAM before diagnosis, with the number of patients using CAM increasing to 61.0% (*n* = 199) after diagnosis. The frequency of each CAM modality used by participants, before and after diagnosis, is presented in Table 3. Nutritional supplements were most commonly used both, before and after diagnosis. The CAM modalities that showed a significant increase in the proportion of people using them after diagnosis were nutritional supplements, Chinese herbal medicines, diet modifications, manipulative-based therapies, biofield therapy, bioelectromagnetic-based therapies, supernatural healing therapies, and mind-body therapies. By contrast, the proportions of people using acupuncture, cupping and scraping, and aromatherapy were not found to be different in the groups before and after diagnosis. 

Use of multiple combinations of CAM (between two and twelve modalities) was reported by 11.0% of participants before diagnosis with Type 2 diabetes and 55.2% after diagnosis. A highly significant difference was found in the mean numbers of modalities used before (mean = 0.48) and after (mean = 2.20) diagnosis (*T*(324) = −11.73, *P* < .001). The result indicates that people with Type 2 diabetes reported more use of CAM after being diagnosed with diabetes.

### 3.3. The Reasons for CAM Use

For each CAM, users were asked to differentiate between their use of CAM to manage their diabetes, to manage diabetic complications, or for others reasons. Of the participants who used CAM, only 24.9% used CAM to control diabetes directly and only 3.2% used CAM to treat diabetic complications; the majority (71.9%) used CAM for other health-related conditions. Although the majority used CAM for reasons other than to treat diabetes and its complications, they did expect some benefits in relation to diabetes from the usage. The most common expectation of the benefits, ranked highest to lowest, were as follows: to reduce symptoms (51.3%), to maintain body health (47.2%), to improve energy (25.4%), to increase metabolism (17.3%), and others, such as to help body self-healing, to improve emotional well-being, to take fewer conventional medicines, and to cure other diseases.

### 3.4. Patients' Experience of Decision-Making about CAM Use

**Figure 1:**
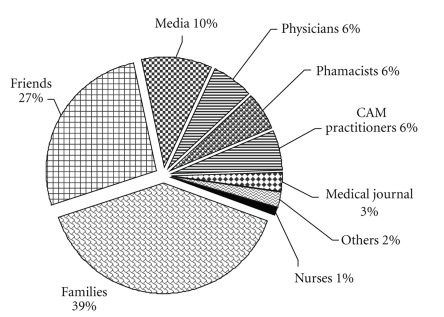
Information sources of CAM used by participants.

Of the after-diagnosis users (*n* = 199) identified from the survey respondents, 197 users completed this section. The majority initially chose CAM because people around them believed in CAM (49.2%) (see Table 4). The primary source of CAM information was families (49.2%) and friends (33.5%), with only 3% identifying CAM practitioners as the primary information source (see Figure 1). However, the main decision on CAM use was still taken by the participants themselves (75.6%), while only 18.3% reported that the decision to use CAM was made by family members and 2% reported that the decision was made by friends. 

### 3.5. CAM Knowledge among Users

A surprising 63.4% of after-diagnosis users of biologically based therapies (*n* = 172) reported that they did not know which complementary medicine they were actually taking. Within this group, 26.6% did not know anything about their CAM products, 30.3% could identify that their CAM products came from CAM practitioners, and 43.1% knew that the ingredients were stated on the label. Of 172 participants, only 30.8% did not change the time at which they administered their conventional medication. However, 60.4% of participants took conventional medication and CAM products at separate times to prevent interactions while 5.8% of participants either reduced the dose of conventional medication without informing conventional healthcare professionals or even stopped conventional medication altogether when they took CAM products.

### 3.6. Decisions Regarding Disclosure of CAM Use

Among 197 participants who completed this section, two participants did not visit a healthcare professional during the time they were using CAM. Of 195 participants who saw a healthcare professional and used CAM along with conventional medicines, only 24.6% had disclosed their CAM use to a healthcare professional. This indicated that the majority reported deciding to take a CAM product concurrently with conventional medicine but without seeking advice from a healthcare professional. The reasons people gave for not informing their doctor about their CAM use, were: (a) that they never thought of it (55.8%); (b) that they feel CAM use is safe, thus there is no need to discuss its use (51.9%); (c) that healthcare professionals do not ask about their CAM use (22.4%); (d) that they think that healthcare professionals will discourage CAM use (7.5%); (e) that they think there is not sufficient time to discuss CAM use (4.1%); and (f) that they think that healthcare professionals do not have an adequate knowledge of CAM (4.1%). 

For the minority (*n* = 48) who informed the healthcare professionals of their CAM use, the responses from healthcare professionals were quite negative and passive, including: (a) stating that it was entirely a matter for the patient and offering no comment about the patient's CAM use (47.9%); (b) warning them of possible side effects of CAM use (8.3%); and (c) discouraging them from taking it (6.3%). Only 15 participants (31.3%) reported that their healthcare professionals encouraged them to use CAM.

## 4. Discussion

In this study, more than three in five people with Type 2 diabetes had used at least one type of CAM after diagnosis, which is consistent with some previous studies [17, 18]; however, this usage is higher than the studies in Malaysia (35.5%) [3], Turkish (41.0%) [19], and Germany (18.4%) [12]. It is important to note that the reported prevalence of CAM use in general varies widely in the literature [4, 20] because research design, methods of data collection, time frames, sample population, response rates, and the operational definition of what constitutes a CAM greatly affect the estimation of CAM use. The high level of CAM use concomitantly with conventional medicines found in this study confirms that patients do not use CAM to replace conventional medicine but rather to complement it. 

The top five CAM modalities most commonly used before diagnosis were nutritional supplements, Chinese herbal medicines, cupping and scraping, acupuncture, and supernatural healing therapies. This pattern slightly changed after diagnosis, with use of manipulative- and body-based therapies and diet modifications significantly increasing after diagnosis whereas acupuncture and cupping and scraping use decreased. These changes may indicate that CAM became a self-care strategy for people with Type 2 diabetes not only to manage their diabetes but also their general health and well-being. The patterns of CAM use after diagnosis were similar to some studies in the USA [21] and Thailand [22] and contrasted with the studies in the UK [5] and India [23] in which naturopathy, ayurveda, homeopathy, reflexology, and aromatherapy were popular with people living with diabetes. While nutritional supplements and diet modification are cross-cultural phenomena, Chinese herbal medicine and supernatural healing therapies are Oriental, and their popularity in Taiwan indicates that traditional culture and religious beliefs heavily influence the selection of CAM therapies. 

Indeed, there are nine (9) therapies for which the usage rate significantly increases after diagnosis with diabetes. These therapies include Chinese herbal medicine, manipulative- and body-based therapies, diet modifications, biofield therapy, and supernatural healings, which are all related to traditional Chinese medicine (TCM). TCM comprises of a wide range of techniques, such as acupuncture, electro-acupuncture, moxibustion, auriculotherapy, cupping, Tui-na (massage), herbal medicine, pharmacology, dietary therapy, Qigong, Taichi, and Feng-shui. The theory of TCM is based on a number of philosophical frameworks including the theory of yin and yang. The treatments listed above are intended to restore an imbalance of yin and yang [24], for example, Chinese dietary therapy is the most basic treatment in TCM which classifies food as either yin or yang or toxic foods [25]. Taiwan is an island off the eastern coast of Asia with a population of twenty-three million people. More than 80% of Taiwanese people are the descendants of immigrants coming from southeast China since the 16th century (Fujianese and Hakka). These immigrants brought traditional Chinese medicine (TCM) to Taiwan; hence, its practitioners have been providing health care service privately to Taiwanese for several centuries. People share these beliefs and common experiences that affect their engagement of behaviours in treating, preventing or modifying illness. 

Since people viewed CAM use as one element in an individually mediated approach to the self-management of their health and illness, it is important for healthcare professionals to be aware of which CAM modalities are commonly used by people after diagnosis with Type 2 diabetes and why. The majority of participants in this study primarily used CAM for other health-related conditions; to relieve symptoms related to conditions other than Type 2 diabetes, to maintain body health, and to improve energy, thus confirming the findings of previous studies [26, 27]. It seems that the majority of those who used CAM considered wellness and quality of life as more important than directly treating diabetes. However, the opinion of others heavily influenced the decision-making process and the most common reason for initial CAM use, reported by participants, was that people close to them believed in the efficacy of CAM. As the main sources of CAM information identified by the participants were informal and unscientific, there may be concern about the quality and safety of CAMs used by people with Type 2 diabetes, especially as they were frequently using CAM together with conventional medicines. In addition, limited disclosure of CAM use to their healthcare professionals, and a negative or passive reaction from the healthcare professionals upon disclosure complicates the situation which may result in inadequate treatment, decreased health outcomes, and higher costs for the health care system.

## 5. Implications

Awareness of widespread use of CAM modalities by people with Type 2 diabetes is crucial for healthcare professionals. Our study indicated people with Type 2 diabetes often use unreliable CAM information sources, have a lack of knowledge about the CAM products they are taking, and self-administer both types of medicines concomitantly. Healthcare professionals need to acknowledge CAM use, learn to discuss CAM use with their patients, and be able to do so in an open-minded, respectful manner. Moreover, healthcare professionals need to be able to provide accurate evidence-based advice about CAM. This information will be crucial to health professionals in providing effective care and needs to be incorporated into professional education and research, as well as clinical practice. Education about CAM, however, should not only focus on healthcare professional education but should also consider patient education. This study has highlighted concerns about patients' decision-making processes related to CAM use, including poor quality information sources and lack of guidance. Therefore, up-to-date evidence-based CAM information should be provided to patients as part of diabetes routine management and counselling. Given that the opinions of others profoundly influenced patients' decisions about CAM use, patient education about CAM should also involve patients' social networks such as family. It is therefore important to empower patients with evidence-based information and integrate CAM modalities into chronic disease self-management programs. This shift towards a holistic model of care will provide optimal patient care in the future.

## 6. Limitations

The design involved retrospective data collection in which recall bias can not be excluded, especially regarding CAM use before and after the diagnosis of diabetes. The use of cardboard prompt list with the names and descriptions of the various CAM modalities was used assist with recall. Additionally, if recall was low, it was probably similarly low across the sample because there are no obvious reasons to expect differential reporting of CAM use compared to other previous Taiwanese studies. A second limitation is sampling bias. Cluster sampling tends to be less accurate than other samplings. Therefore, to avoid sample error, the discrepancy between sample characteristics and the true population characteristics were compared and showed homogeneity between the two groups. The nonresponse rate in this study also remained low, which minimized the sampling bias.

## 7. Conclusion

The majority of people with Type 2 diabetes in Taiwan are likely to use both conventional medicine and CAM in managing their illness and health without using appropriate information sources to support their decision or disclosing their CAM use to their healthcare professional. Although evidence is mounting in support of the use of various CAM to treat a wide variety of complications of diabetes, whether patients with Type 2 diabetes actually use CAM with known benefits in the management of diabetes is largely unknown. A significant increase in the usage of Chinese herbal medicines, nutritional supplements and non-Chinese herbal medicines following diagnosis with Type 2 diabetes and the unsupervised use of these CAM modalities with conventional medicines bears the risk of CAM-drug interactions which may compromise the optimal management of diabetes. The findings in this study can be used to improve healthcare professional awareness, patient assessment, healthcare professional and patient education, and clinical research. Improving CAM education for patients and healthcare professionals and developing an open and honest communication between patients and healthcare professionals is imperative for effective patient care when managing a chronic disease.

## Figures and Tables

**Table 1:** Cardboard prompt list for conducting the face-to-face interviews with the participants explaining the content of fourteen CAM therapies.

Items	CAM modalities	Specific treatments
1	Acupuncture	To insert fine needles into acupoints, moxibustion, and acupressure
2	Homeopathy	Animal, plant, mineral, and synthetic substances in its remedies, isopathy, and flower remedies
3	Chinese herbal medicine	Ginseng (Panax ginseng), Dong quai (Angelica sinensis), and Licorice (Glycyrrhiza glabra)
4	Nutrients supplements	Multiple-vitamins, vitamins, fish oil, minerals, and glucosamine
5	Non-Chinese herbs	Bilberry, opunita, and fenugreek seed
6	Diet modification	Organic food, purified diet (not suggested by conventional healthcare professionals)
7	Cupping, Scraping	Cupping: the cup to stick to the skin via suction, Gua-sha, and Tui-na
8	Manipulative-based therapies	Chiropractic, osteopathic, and kneading
9	Folk therapies	Knife therapy, water therapy, and fire therapy
10	Biofield therapy	The human body kinematics, Gi-gone, Tai-chi, and Reiki,
11	Bioelectromagnetic-based therapies	Magnetic fields, pulsed fields, and two polar faradisms
12	Supernatural healings	Shou-jing, ji-tong, fengshui, bai-bai, divination, and changing individual's name
13	Aromatherapy	Aroma oil, balsam, lavender, and peppermint oil
14	Mind-body therapy	Meditation, yoga, and hypnotization

**Table 2:** Demographic characteristics of the overall sample (*n* = 326).

Sample characteristics	Percent
Gender	
Male	44.2
Female	55.8

Age (years)	
18–44	12.3
45–54	29.1
55–64	31.6
≥65	27.0

Race	
Fujan	63.8
China	22.7
Haka	11.0
Aboriginal	7.5

Highest education	
< High school graduate	46.9
≥ High school graduate	53.1

House income	
≤ US$ 10,000 (NT$330,000)	19.0
US$ 10,001–30,000 (NT$330,001–990,000)	51.5
≥ US$ 30,001 (NT$990,001)	24.0
Unknown	5.5

Duration of diagnosis (years)	
1–5	48.5
6–10	30.4
≥10	21.1

Diabetes treatment	
Oral agent	85.6
Insulin	9.8
Both	4.6

Clinic visit frequency	
≤ Monthly	81.3
> Monthly	18.7

**Table 3:** Comparative frequency of use of CAM modalities before- and after-diagnosis with Type 2 diabetes.

CAM modalities	Before diagnosis %	After diagnosis %	*P* value
Whole medical systems			
Acupuncture	5.5	6.7	.60
Homeopathy	0.0	0.0	NA

Biologically based practices			
Chinese herbal medicines	8.0	27.9	<.001
Nutritional supplements	8.6	41.1	<.001
Diet modification	1.8*	13.2	.003
Non-Chinese herbs	0.3*	3.4	.006

Manipulative- and body-based practices			
Cupping, scraping	5.8	6.4	.84
Manipulative-based therapies	4.6	13.5	<.001
Folk therapies	0.3*	0.6	1.000

Energy medicine			
Biofield therapy	1.8*	9.2	<.001
Bioelectromagnetic-based therapies	3.7	10.1	.001

Mind-body medicine			
Supernatural healings	4.9*	11.0	<.001
Mind-body therapies	1.5*	3.7	.04
Aromatherapy	1.2*	0.3*	.25

*Cells have expected count less than 5.

**Table 4:** The reasons for starting CAM use.

Initial reasons for CAM use	*n* = 197	%^1^
People around them believed in CAM treatment	97	49.2
They believed in CAM	76	38.6
CAM was consistent with their culture	23	11.7
CAM was perceived to have fewer side effects than conventional medicine	20	10.1
CAM was recommended by healthcare professionals	14	7.1
They were dissatisfied with conventional medicine	2	1.0

^1^The total percentage is greater than 100% because the multiple responses were allowed in the question.

## Abbreviations

CAM: Complementary and Alternative Medicine
SPSS: Statistics Package for Social Science
TCM: Traditional Chinese Medicine
UK: United Kingdom
USA: United States of America.
